# Modelling of microbial polyhydroxyalkanoate surface binding protein PhaP for rational mutagenesis

**DOI:** 10.1111/1751-7915.12820

**Published:** 2017-08-25

**Authors:** Hongyu Zhao, Zhenyu Yao, Xiangbin Chen, Xinquan Wang, Guo‐Qiang Chen

**Affiliations:** ^1^ Center for Synthetic and Systems Biology School of Life Sciences Tsinghua‐Peking Center for Life Sciences Tsinghua University Beijing China; ^2^ MOE Laboratory of Protein Science Beijing Advanced Innovation Center for Structural Biology Collaborative Innovation Center for Biotherapy School of Life Sciences Tsinghua University Beijing 100084 China; ^3^ Collaborative Innovation Center for Biotherapy State Key Laboratory of Biotherapy and Cancer Center West China Hospital West China Medical School Sichuan University Chengdu China; ^4^ Manchester Institute of Biotechnology University of Manchester Manchester UK; ^5^ Center for Nano and Micro‐Mechanics Tsinghua University Beijing 100084 China; ^6^ MOE Key Lab for Industrial Biocatalysis Tsinghua University Beijing 100084 China

## Abstract

Phasins are unusual amphiphilic proteins that bind to microbial polyhydroxyalkanoate (PHA) granules in nature and show great potential for various applications in biotechnology and medicine. Despite their remarkable diversity, only the crystal structure of PhaP_*A*__*h*_ from *Aeromonas hydrophila* has been solved to date. Based on the structure of PhaP_*A*__*h*_, homology models of PhaP_*A*__*z*_ from *Azotobacter sp*. FA‐8 and PhaP_*TD*_ from *Halomonas bluephagenesis* TD were successfully established, allowing rational mutagenesis to be conducted to enhance the stability and surfactant properties of these proteins. PhaP_*A*__*z*_ mutants, including PhaP_*A*__*z*_Q38L and PhaP_*A*__*z*_Q78L, as well as PhaP_*TD*_ mutants, including PhaP_*TD*_Q38M and PhaP_*TD*_Q72M, showed better emulsification properties and improved thermostability (6‐10°C higher melting temperatures) compared with their wild‐type homologues under the same conditions. Importantly, the established PhaP homology‐modelling approach, based on the high‐resolution structure of PhaP_*A*__*h*_, can be generalized to facilitate the study of other PhaP members.

## Introduction

Polyhydroxyalkanoates (PHA) are polymers that are accumulated as intracellular carbon and energy storage compounds in many bacteria (Anderson and Dawes, 1990; Madison and Huisman, 1999; Keshavarz and Roy, 2010). The stored PHA helps the bacteria survive extreme conditions such as temperature shifts, changes of pH and osmotic pressure and/or lack of nutrients (Potter, 2004; Wang *et al*., 2014). Due to their excellent biodegradability, biocompatibility and thermo‐processability, PHAs are not only interesting as environmentally friendly bioplastics (Chen *et al*., 2001; Potter *et al*., 2001), but also as biomaterials suitable for implant purposes and as drug delivery matrices (Potter, 2004; Wang *et al*., 2014).

PHAs are accumulated as intracellular granules surrounded by an organized protein layer composed of PHA synthases, PHA depolymerases and the amphiphilic phasins or PhaPs, which are the object of this study (Potter and Steinbuchel, 2005; Jendrossek and Pfeiffer, 2014). The combination of structural, synthetic and catabolic proteins on the surface of PHA granules forms the complex structure of the carbonosome (Potter and Steinbuchel, 2005; Jendrossek and Pfeiffer, 2014). It is well established that PhaPs bind to the surface of PHA granules (Pfeiffer and Jendrossek, 2011, 2012), forming an interphase between the cytoplasm and the hydrophobic PHA granules to prevent granular coalescence (Wieczorek *et al*., 1995). A number of studies have demonstrated potential applications of PhaPs in biotechnology and medicine, from protein purification, surface coating and enzyme chaperones to targeted drug delivery (Qi *et al*., 2000; Handrick *et al*., 2004; Kuchta *et al*., 2007; Galan *et al*., 2011). In view of this, it has become increasingly attractive to understand the structure and function of PhaPs. Apart from their structural role on the surface of PHA granules, other PhaP functions have been reported, including influencing the number and sizes of PHA granules and promoting PHA accumulation (Wieczorek *et al*., 1995; Kuchta *et al*., 2007). Some PhaPs were reported to have regulatory activity, such as PhaF from *Pseudomonas putida*, which regulates PHA synthesis (Prieto *et al*., 1999; Galan *et al*., 2011), ApdA from *Rhodospirillum rubrum*, which induces polyhydroxybutyrate (PHB) depolymerization (Handrick *et al*., 2004), PhaP_1_ of *R. eutropha*, which enhances the specific activity of PHA polymerase *in vivo* (Qi *et al*., 2000), and PhaP_*Ah*_ from *Aeromonas hydrophila* strain 4AK4, which affects its cognate PHA polymerase at different levels (Tian *et al*., 2005). The effects of PhaPs on PHA accumulation are attributed to several mechanisms, including the activation of genes involved in PHA synthesis (Tian *et al*., 2005) and the formation of a barrier between the PHA granules and other intracellular components to help avoid negative effects of PHA accumulation on cellular activities (Wieczorek *et al*., 1995).

Although the degree of conservation among PhaPs is very low, some motifs that are characteristic of most if not all PhaPs have also been described (Mezzina *et al*., 2014). Based on alignments using the Pfam database, four phasin‐related families have been found (Finn *et al*., 2014). The first family, termed Phasin_2 (PF09361), is the largest and includes 952 sequences found in bacteria belonging to *Alpha‐*,* Beta‐* and *Gammaproteobacteria*. These include PhaP1 from *Ralstonia eutropha*, PhaP_*Ah*_ from *Aeromonas hydrophila*, PhaP_*Az*_ from *Azotobacte**r*** and PhaP_*TD*_ from *Halomonas bluephagenesis* TD. The second PhaP family (PF09602) is related to phasins found in *Bacillus* species, and the third one (PF09650) contains a diverse group of mostly uncharacterized proteins found in different *Proteobacteria*. The last one (PF05597) consists of proteins from different *Proteobacteria*, including the most typical phasins belonging to *Pseudomonas* spp., such as PhaF and PhaI from *P. putida* (Mezzina and Pettinari, 2016).

A small group of PhaPs has been investigated in greatest detail: PhaP_*Ah*_ from *Aeromonas hydrophila* strain 4AK4 has been employed as a protein purification tag that binds to PHA particles for easy recovery, as a drug delivery tool, as a coating agent and as a biosurfactant (Moldes *et al*., 2004; Banki *et al*., 2005; Backstrom *et al*., 2007; Wang *et al*., 2008). The PhaP‐based protein purification system was successfully used to inexpensively purify enhanced green fluorescent protein (eGFP), maltose‐binding protein and β‐galactosidase (Wang *et al*., 2008). Additionally, PhaP_*Ah*_ has been shown to be a highly efficient biosurfactant (Wei *et al*., 2011). In a further potentially groundbreaking application, PhaP_*Ah*_ fused with targeted cell ligands has been reported to specifically bind to cancer cells (Yao *et al*., 2008). PhaP_*Az*_ from *Azotobacter sp*. FA‐8 is reported to not only improve growth and PHB accumulation in recombinant *Escherichia coli*, but also protect non‐PHB synthesizing *E. coli* under both normal and stress conditions, as evidenced by a reduction in heat‐shock protein levels, leading to better growth and stronger resistance to both heat shock and superoxide stress (de Almeida *et al*., 2007, 2011). PhaP_*Az*_ was also proven to help protein folding by preventing spontaneous thermal aggregation in a chaperone‐like manner, both *in vivo* and *in vitro* (Mezzina *et al*., 2014). Due to the many potential applications, research into industrial production of phasins is also ongoing. For example, *Halomonas bluephagenesis* TD01 was used for high yield expression of its own PhaP_*TD*_ in an economical way (Lan *et al*., 2016), and it was found to be a suitable biosurfactant.

Even though numerous phasins have been characterized, only the structure of PhaP_*Ah*_ has been solved. It was shown to be a tetramer with 8 α‐helices adopting a coiled‐coil structure, with each monomer having a hydrophobic and a hydrophilic surface, which explains its surfactant properties (Zhao *et al*., 2016). Although PhaP_*Az*_ and PhaP_*TD*_ both have remarkable properties with interesting biotechnological applications, no study has been conducted to clarify the corresponding structures, other than theoretical predictions of their secondary structure (Mezzina *et al*., 2014).

To address this issue, we established homology models of PhaP_*Az*_ and PhaP_*TD*_ based on the crystal structure of PhaP_*Ah*_, which allowed us to reveal the biochemical and functional mechanisms of these two PhaPs. Rational mutations were designed based on these homology models, and the enhanced PhaP stability and surfactant properties of the resulting mutant proteins confirmed the validity of the structures. We hope that this publication will provide a generalized method for the homology modelling of PhaP structures to facilitate further studies.

## Results

### Secondary structure analysis of PhaP_*Az*_ and PhaP_*TD*_

The PhaP_*Az*_ and PhaP_*TD*_ proteins were successfully expressed in *E. coli* BL21 (DE3) and *Halomonas bluephagenesis* TD01 respectively. They were purified using affinity‐ and size‐exclusion chromatography, and their purity confirmed using SDS‐PAGE (Fig. S1). Static light scattering (SLS) studies indicated that the average molecular weight of PhaP_*Az*_ was 90 kDa (Mezzina *et al*., 2014). As the PhaP_*Az*_ monomer weight was predicted to be 21 kDa, and based on their overall similarity, we expected both PhaPs to be tetramers in aqueous solution. However, SLS revealed the molecular weight of PhaP_*TD*_ to be 48 kDa (Fig. S2). As the predicted molecular weight for a PhaP_*TD*_ monomer is approximately 15 kDa, PhaP_*TD*_ may be a trimer in solution. How these two PhaPs evolved different oligomerization remains to be elucidated.

**Figure 1:**
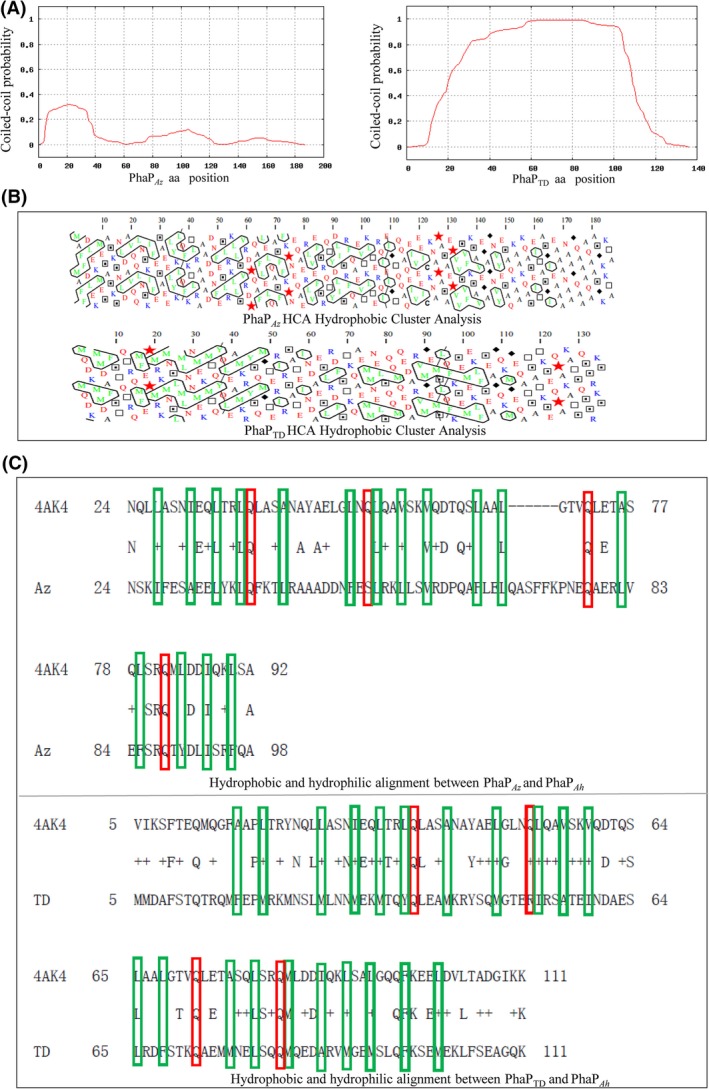
Secondary structure analysis of PhaP_*A*__*z*_ and PhaP_*TD*_. (A) Probability of coiled‐coil regions as determined using MARCOIL. (B) Hydrophobic cluster analysis. The green letters circled by black line are hydrophobic cluster. The red stars are marked to highlight the potential hydrophobic cluster. The blue letters are strong hydrophilic amino acids, and the red letters are also hydrophilic residues less than blue ones. (C) Amphiphilic character alignments among PhaP_*A*__*z*_, PhaP_*TD*_ and PhaP_*A*__*h*_. 4AK4 denotes PhaP_*A*__*h*_ from *Aeromonas hydrophila* strain 4AK4, *Az* denotes PhaP_*A*__*z*_ from *Azotobacter sp*. FA‐8, and TD denotes the PhaP_*TD*_ from *Halomonas bluephagenesis* TD01. Green blocks mark the hydrophobic amino acids on the hydrophobic surface of PhaP_*A*__*h*_. Red blocks mark the hydrophilic amino acids on the hydrophobic surface of PhaP_*A*__*h*_.

*In silico* predictions of phasin secondary structures have found a general feature of phasins – a high percentage of amino acids arranged in an α‐helix conformation (Mezzina *et al*., 2014), together with coiled‐coil interacting regions(Maestro *et al*., 2013; Mezzina and Pettinari, 2016). This common structure suggests that the mode of oligomerization could be determined by the coiled‐coil regions. Using MARCOIL (Delorenzi and Speed, 2002; Mezzina *et al*., 2014), PhaP_*Az*_ was found to have a maximum probability of 32% of coiled‐coil regions for homo‐oligomerization, while PhaP_*TD*_ contains a high coiled‐coil probability along its entire sequence, which was similar to PhaP_*Ah*_ (Fig. 1A).

Thus, the oligomerization of both PhaP_*Az*_ and PhaP_*TD*_ may be attributed to coiled‐coil regions similar to PhaP_*Ah*_, as evidenced by the structure predictions. As all phasins share the unique ability of binding to hydrophobic granules, analysis of hydrophobic domains is important. Interestingly, hydrophobic cluster analysis (HCA) of PhaP_*Az*_ and PhaP_*TD*_ showed that these two proteins do not possess clear hydrophobic domains (Fig. 1B) (Mechin *et al*., 1995). In fact, hydrophobicity‐modulating mutations of PhaP_*Re*_ were reported to implicate the entire protein in interactions with the PHA polymer granules, without a distinct region responsible for this interaction (Neumann *et al*., 2008). Nevertheless, judging by the only resolved crystal structure of a phasin (Zhao *et al*., 2016), a clear distribution of hydrophilic and hydrophobic residues is found on two opposite sides of the PhaP_*Ah*_ monomer, forming an intrinsically amphiphilic polypeptide without a distinct hydrophobic core (Zhao *et al*., 2016). These clues suggest that PhaP_*Az*_ and PhaP_*TD*_ may possess the same pattern as was observed in PhaP_*Ah*_. A sequence alignment has been conducted to find similarities among PhaP_*Az*_, PhaP_*TD*_ and PhaP_*Ah*_ (Fig. 1C). However, a clear sequence homology could not be found even though their amphiphilic properties had a similar pattern. Nevertheless, blocks of hydrophobic amino acids on the *bona fide* hydrophobic surface of PhaP_*Ah*_ (shown in green, Fig. 1C) were reflected in hydrophobic amino acids in the corresponding positions of PhaP_*Az*_ and PhaP_*TD*_. At the same time, hydrophilic amino acids were found in the hydrophobic surface of PhaP_*Ah*_ (shown in red, Fig. 1C), as was also found in PhaP_*Az*_ and PhaP_*TD*_. A majority of these hydrophilic residues were conserved glutamines.

### Rational mutagenesis based on the homology models of PhaP_*Az*_ and PhaP_TD_

**Figure 2:**
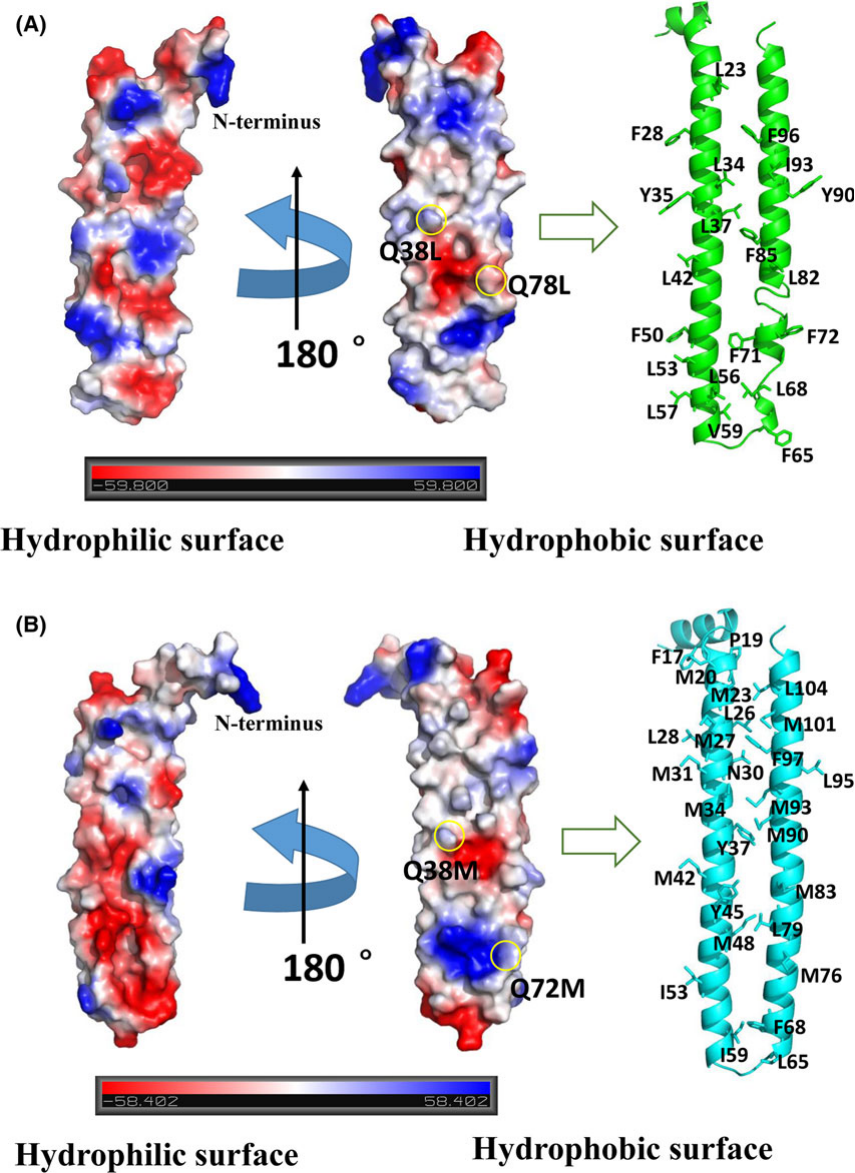
Homology modelling of PhaP_*A*__*z*_ and PhaP_*TD*_ and rational mutagenesis based on the obtained structures. (A) Amphiphilic surfaces of PhaP_*A*__*z*_. The hydrophobic amino acids on the *bona fide* hydrophobic surface are shown in green. The two rational mutations are indicated with yellow circles. (B) Amphiphilic surfaces of PhaP_*TD*_. Hydrophobic amino acids are shown in cyan. The two rational mutations are indicated with yellow circles.

Based on secondary structure analysis of PhaP_*Az*_ and PhaP_*TD*_, a strong structural similarity was found between these two proteins and PhaP_*Ah*_. Accordingly, homology models of these proteins were established based on the crystal structure of PhaP_*Ah*_ using SWISS‐MODEL (Fig. 2A and B)(Biasini *et al*., 2014). These two modelled structures clearly demonstrated an intrinsic amphiphilic property, even though they did not show clear hydrophobic domains. A very significant phenomenon was observed in both structures via surface electrostatic potential analysis, regarding the distribution of hydrophilic and hydrophobic residues on two opposite sides of individual PhaP_*Az*_ and PhaP_*TD*_ monomers (Fig. 2A and B). There are 20 hydrophobic amino acids forming the hydrophobic surface of PhaP_*Az*_ (Fig. 2A). Most of these residues are leucines and phenylalanines, with hydrophobic residues also found in the corresponding positions of PhaP_*Ah*_ (Fig. 1C). By contrast, there are 27 hydrophobic amino acids forming the hydrophobic surface of PhaP_*TD*_ (Fig. 2B), and most of these residues are methionines. The corresponding positions are also covered by hydrophobic residues in PhaP_*Ah*_ (Fig. 1C). It seems that most of the hydrophobic amino acids are folded on the same layer to form a hydrophobic surface through conformational changes of the α‐helices. This may be the reason why phasins have the ability to bind to PHA inclusion bodies, even though PhaP primary structures lack clear hydrophobic domains.

Based on the structural information of PhaP_*Az*_ and PhaP_*TD*_, two significant hydrophilic amino acids embedded in the hydrophobic surfaces were identified as targets for rational mutagenesis of each monomer, which may improve their stability and amphiphilic properties (Fig. 2A and B). The hydrophilic glutamines Q38 and Q78 of PhaP_*Az*_ were mutated to hydrophobic leucines (Fig. 2A) – the most abundant hydrophobic residues in this structure. Subsequently, the hydrophilic glutamines Q38 and Q72 of PhaP_*TD*_ were mutated to hydrophobic methionines (Fig. 2B), which again were the most abundant hydrophobic residues in the second structure. The two mutant proteins were successfully expressed and purified from *E. coli*. These mutations might be able to enhance the stability of their respective tetramers by increasing hydrophobic interactions, as was observed in PhaP_*Ah*_(Zhao *et al*., 2016). Furthermore, our aim was also to obtain enhanced emulsification properties by introducing additional hydrophobic amino acids into the hydrophobic surface.

### Rational mutations of PhaP_*Az*_ and PhaP_*TD*_ resulted in improved stability

**Figure 3:**
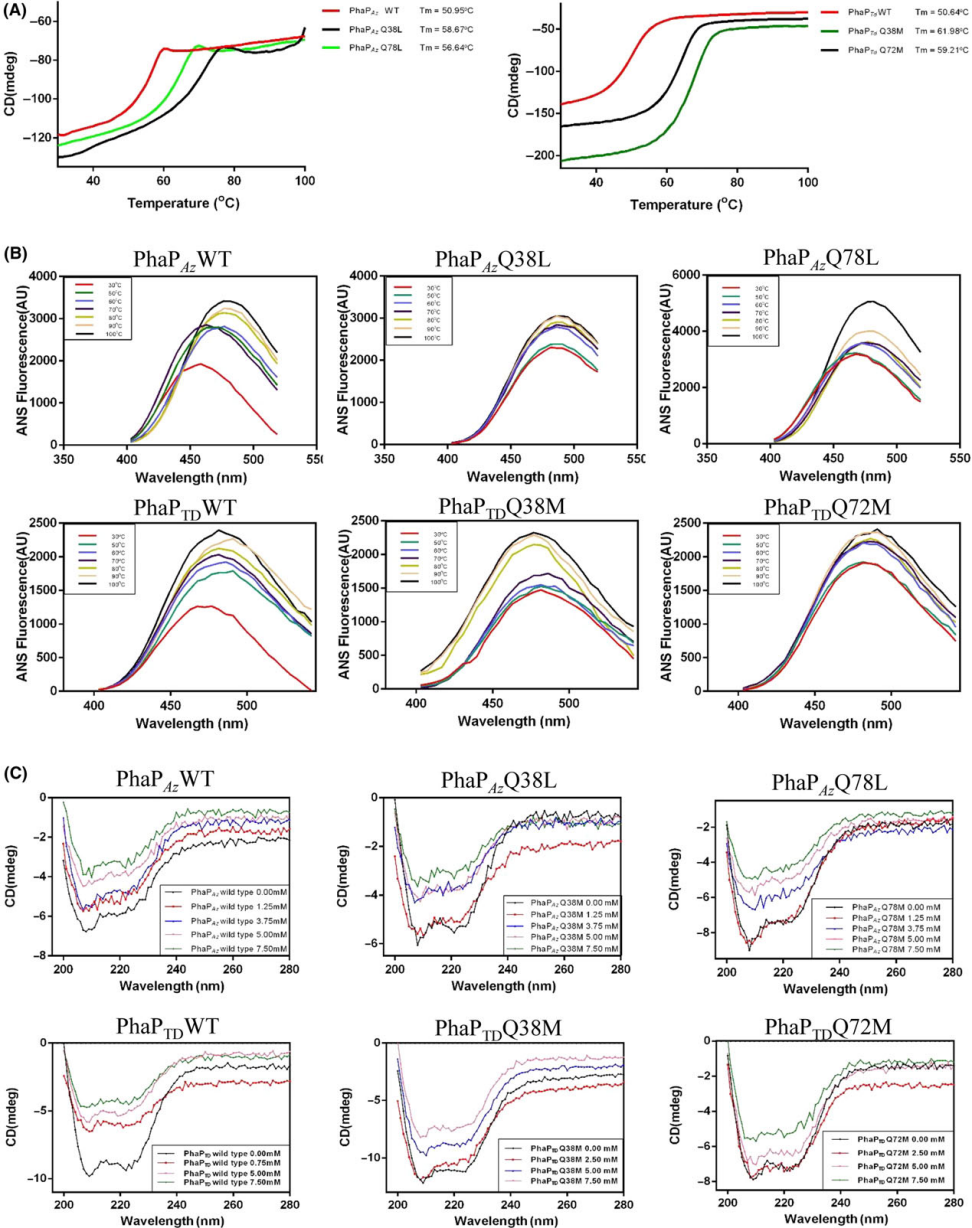
Improvement of thermal stability by rational mutations of PhaP_*A*__*z*_ and PhaP_*TD*_. (A) Melting temperatures of wild‐type and mutant PhaP_*A*__*z*_ and PhaP_*TD*_, studied by circular dichroism (CD) spectroscopy. Thermal graphs were recorded between 30 °C and 100 °C. (B) Hydrophobic exposure of wild‐type and mutant PhaP_*A*__*z*_ and PhaP_*TD*_ at different temperatures studied using 1‐anilinonaphthalene‐8‐sulfonate (ANS) fluorescence. (C) Improved secondary structure stability of wild‐type and mutant PhaP_*A*__*z*_ and PhaP_*TD*_ measured by CD spectroscopy. CD spectra of wild‐type and mutant PhaP_*A*__*z*_ and PhaP_*TD*_(0.1 mg/ml) in emulsions ranging from 0 to 10 mM sodium oleate in water.

Protein melting temperatures (T_m_) were used to study the thermostability of the rational mutants and their respective wild‐type parents. The PhaP proteins were gradually heated to 100°C and thermal denaturation was monitored using circular dichroism (CD) spectroscopy to obtain the melting temperatures (T_m_)_._ Two PhaP_*Az*_ mutants showed higher T_m_ values than the wild type, with approximately 8 degree increase for PhaP_*Az*_Q38L and 6 degree increase for PhaP_*Az*_Q78L (Fig. 3A). At the same time, significant thermostability increases were observed for PhaP_*TD*_ mutants, with approximately 10 degree higher T_m_ values for both PhaP_*TD*_Q38M and PhaP_*TD*_Q72M (Fig. 3A). Importantly, an analysis of the mutants' 1‐anilinonaphthalene‐8‐sulfonate (ANS) binding affinity also showed similar results (Fig. 3B). The circular dichroism results have shown that these proteins still kept some secondary structures rather than complete melting at 100°C (Fig. S3).Wild‐type PhaP_*Az*_ exposed its hydrophobic sites at 50°C, indicating melting at this temperature. On the other hand, its derived mutants PhaP_*Az*_Q38L and PhaP_*Az*_Q78L did not show any significant changes at 50°C, and rapid melting was evidenced only at 60°C (Fig. 3B). Similarly, hydrophobic sites of wild‐type PhaP_*TD*_ were exposed at 50°C, while the mutants PhaP_*TD*_ Q72M and PhaP_*TD*_ Q38M showed significant changes only at 60°C and 70°C respectively (Fig. 3B). Therefore, significantly enhanced thermal stability was achieved through targeted mutations of the two PhaPs. The observed T_m_ differences are most likely due to closer hydrophobic chain interactions.

As phasins are commonly located on the hydrophobic surface of PHA granules, PhaP structures may be affected by hydrophobic environments, and PhaP_*Az*_ has already been proved to change its secondary structure in the presence of sodium oleate (Mezzina *et al*., 2014). It was estimated that approximately 40–45% of the residues in PhaP_*Az*_ were disordered when it was not bound to a target, and this value decreased to between 23 and 30% upon interaction with sodium oleate (Mezzina *et al*., 2014). This decrease of disordered secondary structures may be caused by the change of PhaP oligomers. As PhaP_*Ah*_ does not have obvious hydrophobic regions exposed to the solvent in an aqueous solution (Zhao *et al*., 2016)*,* PhaP_*Ah*_ and PhaPs in general need to change their structure to expose their hydrophobic surfaces to contact PHA. The change of oligomers appears as a plausible way to achieve this. When sodium oleate was used as a triggering substance to study the oligomer stability of PhaPs, the secondary structure of wild‐type PhaP_*Az*_ changed in the presence of 8.5 mM sodium oleate, whereas the mutants PhaP_*Az*_Q38L and PhaP_*Az*_Q78L maintained their secondary structures at concentrations of up to 10 mM sodium oleate, and their conformations changed significantly only at 12.5 mM sodium oleate (Fig. 3C). On the other hand, the secondary structure of wild‐type PhaP_*TD*_ changed at 0.75 mM sodium oleate, whereas its mutants PhaP_*TD*_Q38M and PhaP_*TD*_Q72M tolerated 2.50 mM sodium oleate (Fig. 3C). These results further demonstrate that the mutants were able to withstand higher sodium oleate concentrations than the wild type, clearly illustrating that the targeted mutations improved the proteins' conformational stability in hydrophobic environments by enhancing the hydrophobic interactions between monomer chains, as predicted.

### Rational mutations of PhaP_*Az*_ and PhaP_*TD*_ resulted in improved emulsification properties

**Figure 4:**
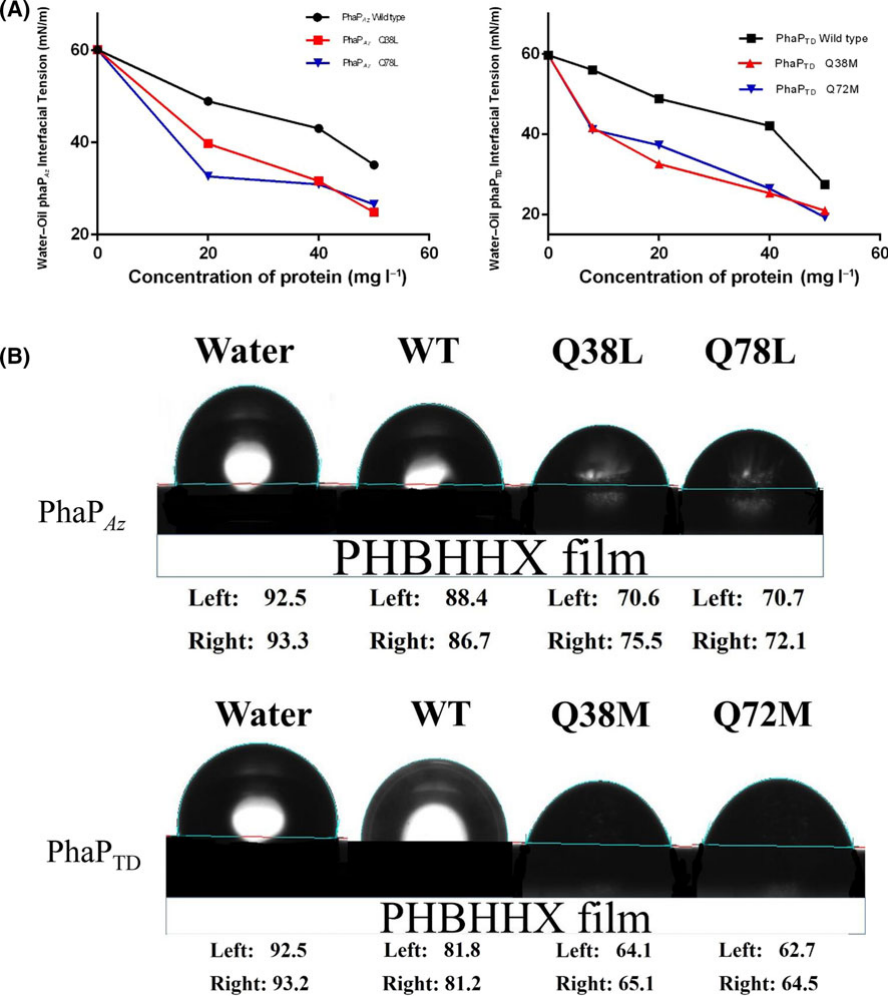
Improvement of emulsification properties by rational mutations of PhaP_*A*__*z*_ and PhaP_*TD*_. (A) Water–oil interfacial tension in the presence of various concentrations of wild‐type PhaP_*A*__*z*_ and PhaP_*TD*_ and the indicated mutants. The water–oil tension between water (PhaP protein solution) and soya bean oil was determined using an optical contact angle measurement and contour analysis system. (B) Comparison of contact angles of drops comprising 25mg/L protein solutions of wild‐type PhaP_*A*__*z*_ and PhaP_*TD*_ and the indicated mutants on hydrophobic PHBHHx films.

Emulsification activity can be measured by analysing a protein's ability to reduce water–oil interfacial tension. The mutants PhaP_*Az*_Q38L and PhaP_*Az*_Q78L showed better properties in reducing water–oil interfacial tension than the wild type at the same protein concentration, demonstrating that an improved emulsification capacity can be obtained by rational mutagenesis (Fig. 4A). The same was also true for PhaP_*TD*_ – its two mutants showed stronger emulsification activity than the parent.

Another way to measure emulsification ability is to study the contact angle of drops comprising aqueous solutions of these proteins placed on hydrophobic surfaces. Equal drops containing the same protein concentrations were carefully applied to a hydrophobic poly(3‐hydroxybutyrate‐co‐3‐hydroxyhexanoate) and a PHBHHx surface, after which the contact angles reflecting the PhaP's amphiphilic properties were recorded. On hydrophobic PHBHHx films, droplets containing the PhaP_*Az*_ mutants Q38L and Q78L had smaller contact angles than droplets containing the wild type (Fig. 4B). Similarly, PhaP_*TD*_Q38M had a smaller contact angle than its wild‐type parent, and PhaP_*TD*_Q72M had the smallest contact angle of all (Fig. 4B). These results indicate improved emulsification ability of the rational mutants compared with their respective wild‐type parents.

## Discussion

Homology models of PhaP_*Az*_ and PhaP_*TD*_ have been constructed successfully for the first time, based on the known crystal structure of PhaP_*Ah*_. The resulting modelled structures enable a rational understanding of the proteins' surfactant properties and the related molecular mechanisms of amphiphilicity. Based on these structures, both PhaP_*Az*_ and PhaP_*TD*_ are predicted to have a hydrophobic and a hydrophilic surface in each monomer (Fig. 2A and B). At the same time, two specific point mutations, for PhaP_*Az*_ and PhaP_*TD*_ each, were rationally designed based on the homology‐modelled structures. In detail, two hydrophilic glutamines and methionines were mutated to hydrophobic leucines in the hydrophobic surfaces of PhaP_*Az*_ and PhaP_*TD*_ respectively (Fig. 2A and B). These designed mutations were expected to improve the stability of PhaP_*Az*_ and PhaP_*TD*_ due to improved hydrophobic interactions between individual chains. As expected, all mutated PhaPs showed increased melting temperatures, indicating enhanced thermal stability (Fig. 3A and B). At the same time, the mutants demonstrated significantly improved conformational stability and emulsification ability under conditions identical to those of their respective wild‐type parents (Figs 3C and 4)**.** Taken together, these results fully support the practical usefulness of the homology‐modelling approach used to generate structural models for PhaP_*Az*_ and PhaP_*TD*_, using the known crystal structure of PhaP_*Ah*_.

Although PhaPs do not have a high degree of sequence conservation, they do contain a high percentage of α‐helices(Zhao *et al*., 2006; Maestro *et al*., 2013; Mezzina *et al*., 2014), together with coiled‐coil interacting regions (Mezzina and Pettinari, 2016). The mode of oligomerization is also dictated by the presence of coiled‐coil regions. Even though the absence of distinct hydrophobic domains appears to be a common phenomenon of most phasins (Mezzina and Pettinari, 2016), the distribution of hydrophilic and hydrophobic residues on two opposite sides of the PhaP_*Az*_ and PhaP_*TD*_ monomers was clearly visible based on the crystal structure of PhaP_*Ah*_ (Fig. 2A and B). The common mechanism of PhaP's amphiphilic properties can thus be attributed to the folding of most of the hydrophobic amino acids surrounding the α‐helical domain in a way conductive to the formation of a hydrophobic surface. Furthermore, the PhaP's ability to bind to hydrophobic PHA or inclusion bodies is also most likely due to this hydrophobic surface.

The proportion of α‐helices in PhaP_*Az*_ was observed to change depending on the environment (Mezzina and Pettinari, 2016). This change was attributed to a change in the proportion of disordered regions. There may be two reasons leading to the change of α‐helix proportion, the first one being the disassembly of PhaP oligomers, as PhaP in an aqueous solution should self‐assemble into oligomers without exposure of its hydrophobic surface, while the presence of hydrophobic molecules would induce PhaPs to disassemble so that their hydrophobic surfaces could bind to these molecules (Zhao *et al*., 2016). The second reason can be attributed to the existence of two different chain conformations, so that some chains may be distorted during the process of oligomer formation (Zhao *et al*., 2016).

PhaPs have also been applied as fusion partners for drug targeting and protein purification (Wang *et al*., 2008; Grage *et al*., 2009), as biosurfactants (Wei *et al*., 2011), and as chaperones for better protein folding(Mezzina *et al*., 2015), positioning PhaPs as candidate proteins for high‐value applications. However, in spite of their impressive functional diversity and biotechnological applications, only the structure PhaP_*Ah*_ has been resolved to date (Zhao *et al*., 2016).

As PhaP structures are important for mechanistic studies and rational mutagenesis, a generalized method was developed in this study to construct PhaP structure models based on the known high‐resolution structure of PhaP_*Ah*_, greatly facilitating the study of other PhaP members, including secondary structure analysis of PhaPs to find amphiphilic features similarity to those of PhaP_*Ah*_. A general PhaP homology‐modelling approach using SWISS‐MODEL can be applied if the PhaP in question has a highly amphiphilic nature similar to PhaP_*Ah*_, which can reasonably be expected in most cases. The results obtained for PhaP_*Az*_ and PhaP_*TD*_ demonstrate the accuracy of this method, paving the way for the analysis of many more PhaPs, hopefully leading to the discovery of new properties and applications.

## Experimental procedures

### Bacterial strains and plasmids

All bacterial strains and plasmids used in this study are summarized in Table 1. *E. coli* S17‐1 was used as donor strain for the *Halomonas* TD conjugation process (Fu *et al*., 2014).

**Table 1:** Bacterial strains and plasmids used in this study

Strain & plasmid	Relevant characteristics	Reference
Strains		
*Escherichia coli* BL21 (DE3)	F^−^ *omp*T hsd S_B_(r_B_^−^ m_B_^−^) *galdcm* (DE3)	Novagen
*E. coli* S17‐1	a vector donor in conjugation, harbours the *tra* genes of plasmid RP4 in the chromosome; *proA, thi‐1*	
*Halomonas bluephagenesis* TD01	*Halomonas* TD wild type, isolated from a salt lake	
Plasmids		
pGEX‐6p‐1	Bacterial expression vector with GST tag, Amp^R^	Tiangene
pSEVA331	A broad‐host vector that can replicate in *Halomonas* TD strains, Cm^R^	

### Vector construction and site‐directed mutagenesis

For protein expression, the gene encoding PhaP_*Az*_ (UniProtKB – Q8KRE9) was cloned into vector pGEX‐6P‐1 and expressed in *E. coli* BL21(DE3) as a fusion protein with an N‐terminal GST tag. The gene encoding PhaP_*TD*_ (UniProtKB ‐ F7SLK4) was inserted into the vector pSEVA331 with a C‐terminal His tag. *E. coli* S17‐1 was selected as the donor for conjugation with *Halomonas bluephagenesis* TD01. Site‐specific mutations were introduced using the Fast Mutagenesis System kit (Transgen Biotech Company).

### Protein expression and purification

The PhaP_*Az*_ protein was expressed in *E. coli* BL21 (DE3) grown in LB medium (10 g/L NaCI, 10 g/L tryptone and 5 g/L yeast extract). A final concentration of 0.5 mM isopropyl‐β‐D‐1‐thiogalactopyranoside (IPTG) was added to the cultures when the OD_600_ reached 0.6–0.8, followed by overnight expression at 37°C. The PhaP_*TD*_ protein was expressed in *Halomonas bluephagenesis* TD01 grown in 60‐LB medium (60 g L^−1^ NaCI, 10 g L^−1^ tryptone and 5 g L^−1^ yeast extract) overnight at 37°C without induction (constitutive expression). Subsequently, cells were harvested by centrifugation at 4000 × g and 4°C for 20 minutes and resuspended in lysis buffer containing 50 mM Tris (pH 8.0), 500 mM NaCl for PhaP_*Az*_, and HBS buffer (1.5 mM Na_2_HPO_4_•2H_2_O, 50 mM HEPS, 150 mM NaCl) for PhaP_*TD*_. After cell disruption, the lysates were centrifuged at 13,000 rpm for 1 h. The cleared lysates containing GST–PhaP_*Az*_ were transferred to a glutathione sepharose 4B column, and the cleared lysates containing PhaP_*TD*_‐His were transferred to a Ni‐NTA agarose column. After washing the column with 5 column volumes (CV) of lysis buffer, the GST‐tagged fusion protein was digested *in situ* using 1 mg/mL 3C PreScission protease, whereas the His‐tagged fusion protein was eluted using elution buffer comprising 500 mM imidazole. The released PhaP_*Az*_ without the GST tag and the His‐tagged PhaP_*TD*_ were further purified by gel‐filtration chromatography on a Superdex 200 high‐performance column using 50 mM Tris–Cl (pH 8.0), 500 mM NaCl and HBS buffer (1.5 mM Na_2_HPO_4_•2H_2_O, 50 mM HEPS, 150 mM NaCl) respectively. The bed volume was 24 mL and the bed dimension was 10×300–310 mm. The injection volume was 1 mL and the flow rate was 0.5 mL min^−1^. The same protocols were applied to the respective mutants.

### *In silico* prediction and homology modelling

MARCOIL was used to predict coiled‐coil regions (Delorenzi and Speed, 2002; Mezzina *et al*., 2014), hydrophobic cluster analysis was used to predict hydrophobic domains (Mechin *et al*., 1995), and homology models based on the crystal structure of PhaP_*Ah*_ were built using SWISS‐MODEL (Biasini *et al*., 2014).

### Protein melting temperature (T_m_) measurements using circular dichroism spectroscopy

Circular Dichroism spectroscopy was carried out on a qCD Chirascan‐auto instrument equipped with the qBiC Biocomparability Suite, using dynamic multimode spectroscopy (DMS). Heat‐induced denaturation of PhaP was directed from 30°C–100°C with a heating rate of 1°C per min. The 220 nm wavelength was used to draw the thermal change graphs. Global 3 software (Applied Photophysics, UK) was used to calculate the melting temperatures. An S curve was applied to fit the data to the following equation (Benjwal, 2006):

y=Ab+{(At−Ab)/(1 + exp[(x_0_‐x)/w]}

whereby y is the experimentally observed CD signal at a given temperature, x is the fraction of the native state at temperature y, Ab, At and w are constant terms of this equation and X_0_ is the transition point of the curve which referring to a protein's melting temperature.

CD measurements were conducted in a buffer comprising 50 mM Tris–Cl (pH 8.0) and 500 mM NaCl for PhaP_*Az*_, and HBS buffer for PhaP_*TD*_. The protein concentration was 0.5 mg/ml for both the wild‐type proteins and the mutants.

### ANS fluorescence spectroscopy

An F‐2500 fluorescence spectrophotometer was used to record fluorescence spectra. The slit width was 5 nm for both emission and excitation. The excitation wavelength was 380 nm for the extrinsic ANS fluorescence. The 1‐sulfonic‐8‐anilinonaphthalene acid (ANS) extrinsic probe was added to the protein solutions at a molar ratio of 75:1 (probe:protein) and incubated at 25°C in the dark for 30 min prior to measurements. ANS binding affinity was measured via emission spectra recorded from 400 to 600 nm. The measurements were conducted in a buffer comprising 50 mM Tris (pH 8.0) and 500 mM NaCl for PhaP_*Az*_, and HBS buffer for PhaP_*TD*_. The protein concentration was 0.5 mg ml^−1^ for both wild type and the mutants.

### Determination of water–oil interfacial tension – pendant drop method

The water–oil tension between water (PhaP protein solution) and soya bean oil was determined using an optical contact angle measurement and contour analysis system (DataPhysics, Germany). The set‐up was used to capture an image of a liquid drop that hangs on a dosing needle and to subsequently analyse it via the DataPhysics SCA 22 software module (DataPhysics, Germany) using the pendant drop method (Berry *et al*., 2015).

### Water contact angle measurements

The OCA20 (the model number of contact angle evaluation system) contact angle evaluation system was applied to measure the water contact angles of protein solution drops which were placed on sample films. Comparison of contact angles of drops comprises 25 mg/L protein solutions of wild‐type PhaP_*Az*_ and PhaP_*TD*_ and the indicated mutants. Droplets with or without protein in 3 μL of deionized water were carefully placed onto poly (3‐hydroxybutyrate‐co‐3‐hydroxyhexanoate) or PHBHHx surfaces. The average contact angles were obtained by measuring drops placed on at least three positions of the same film.

### Static light scattering (SLS)

Protein molecular masses were determined via SLS using the DAWN HELEOSTM II eighteen‐angle static light scattering system (Wyatt Technology, USA) connected to a gel‐filtration chromatography system equipped with a Superdex 200 high‐performance column (see above for specifics of gel filtration). The Superdex 200 high‐performance column was flowed by HBS buffer (1.5 mM Na_2_HPO_4_•2H_2_O, 50 mM HEPS, 150 mM NaCl). The system was pre‐equilibrated with buffer for more than 8 h and subsequently calibrated with 1 mg/ml BSA. The samples were prepared as above and concentrated to 1 mg mL^−1^, after which they were injected into the SLS analyser at a 0.5 mL minutes^−1^ flow rate at 16°C. The molecular mass was calculated using ASTRA5.3.4.14 software (Wyatt Technology, USA).

## Conflict of Interest

The authors have no conflict of interest to declare.

## Supporting information


**Fig. S1.** SDS‐PAGE gels of purified PhaP_*Az*_ and PhaP_*TD*_.
**Fig. S2.** SLS measurements of purified full‐length PhaP_*TD*_.
**Fig. S3.** The circular dichroism results of PhaPAz and PhaP TD at 30°C and 100°C.

## References

[mbt212820-bib-0001] de Almeida, A. , Nikel, P.I. , Giordano, A.M. , and Pettinari, M.J. (2007) Effects of granule‐associated protein PhaP on glycerol‐dependent growth and polymer production in poly(3‐hydroxybutyrate)‐producing Escherichia coli. Appl Environ Microbiol 73: 7912–7916. 17965215 10.1128/AEM.01900-07 PMC2168153

[mbt212820-bib-0002] de Almeida, A. , Catone, M.V. , Rhodius, V.A. , Gross, C.A. , and Pettinari, M.J. (2011) Unexpected stress‐reducing effect of PhaP, a poly(3‐hydroxybutyrate) granule‐associated protein, in Escherichia coli. Appl Environ Microbiol 77: 6622–6629. 21784905 10.1128/AEM.05469-11 PMC3187130

[mbt212820-bib-0003] Anderson, A.J. , and Dawes, E.A. (1990) Occurrence, metabolism, metabolic role, and industrial uses of bacterial polyhydroxyalkanoates. Microbiol Rev 54: 450–472. 2087222 10.1128/mr.54.4.450-472.1990 PMC372789

[mbt212820-bib-0004] Backstrom, B.T. , Brockelbank, J.A. , and Rehm, B.H. (2007) Recombinant Escherichia coli produces tailor‐made biopolyester granules for applications in fluorescence activated cell sorting: functional display of the mouse interleukin‐2 and myelin oligodendrocyte glycoprotein. BMC Biotechnol 7: 3. 17204164 10.1186/1472-6750-7-3 PMC1781935

[mbt212820-bib-0005] Banki, M.R. , Gerngross, T.U. , and Wood, D.W. (2005) Novel and economical purification of recombinant proteins: intein‐mediated protein purification using in vivo polyhydroxybutyrate (PHB) matrix association. Protein Sci 14: 1387–1395. 15883185 10.1110/ps.041296305 PMC2253394

[mbt212820-bib-0006] Benjwal, S. (2006) Monitoring protein aggregation during thermal unfolding in circular dichroism experiments. Protein Sci 15: 635–639. 16452626 10.1110/ps.051917406 PMC2249783

[mbt212820-bib-0007] Berry, J.D. , Neeson, M.J. , Dagastine, R.R. , Chan, D.Y. , and Tabor, R.F. (2015) Measurement of surface and interfacial tension using pendant drop tensiometry. J Colloid Interface Sci 454: 226–237. 26037272 10.1016/j.jcis.2015.05.012

[mbt212820-bib-0008] Biasini, M. , Bienert, S. , Waterhouse, A. , Arnold, K. , Studer, G. , Schmidt, T. , *et al* (2014) SWISS‐MODEL: modelling protein tertiary and quaternary structure using evolutionary information. Nucleic Acids Res 42: W252–W258. 24782522 10.1093/nar/gku340 PMC4086089

[mbt212820-bib-0009] Chen, G.Q. , Zhang, G. , Park, S.J. , and Lee, S.Y. (2001) Industrial scale production of poly(3‐hydroxybutyrate‐co‐3‐hydroxyhexanoate). Appl Microbiol Biotechnol 57: 50–55. 11693933 10.1007/s002530100755

[mbt212820-bib-0010] Delorenzi, M. , and Speed, T. (2002) An HMM model for coiled‐coil domains and a comparison with PSSM‐based predictions. Bioinformatics 18: 617–625. 12016059 10.1093/bioinformatics/18.4.617

[mbt212820-bib-0011] Finn, R.D. , Bateman, A. , Clements, J. , Coggill, P. , Eberhardt, R.Y. , Eddy, S.R. , *et al* (2014) Pfam: the protein families database. Nucleic Acids Res 42: D222–D230. 24288371 10.1093/nar/gkt1223 PMC3965110

[mbt212820-bib-0012] Fu, X.Z. , Tan, D. , Aibaidula, G. , Wu, Q. , Chen, J.C. , and Chen, G.Q. (2014) Development of Halomonas TD01 as a host for open production of chemicals. Metab Eng 23: 78–91. 24566041 10.1016/j.ymben.2014.02.006

[mbt212820-bib-0013] Galan, B. , Dinjaski, N. , Maestro, B. , de Eugenio, L.I. , Escapa, I.F. , Sanz, J.M. , *et al* (2011) Nucleoid‐associated PhaF phasin drives intracellular location and segregation of polyhydroxyalkanoate granules in Pseudomonas putida KT2442. Mol Microbiol 79: 402–418. 21219460 10.1111/j.1365-2958.2010.07450.x

[mbt212820-bib-0014] Grage, K. , Jahns, A.C. , Parlane, N. , Palanisamy, R. , Rasiah, I.A. , Atwood, J.A. , and Rehm, B.H.A. (2009) Bacterial Polyhydroxyalkanoate Granules: Biogenesis, Structure, and Potential Use as Nano‐/Micro‐Beads in Biotechnological and Biomedical Applications. Biomacromol 10: 660–669. 10.1021/bm801394s 19275166

[mbt212820-bib-0015] Handrick, R. , Reinhardt, S. , Schultheiss, D. , Reichart, T. , Schuler, D. , Jendrossek, V. , and Jendrossek, D. (2004) Unraveling the function of the Rhodospirillum rubrum activator of polyhydroxybutyrate (PHB) degradation: the activator is a PHB‐granule‐bound protein (phasin). J Bacteriol 186: 2466–2475. 15060050 10.1128/JB.186.8.2466-2475.2004 PMC412128

[mbt212820-bib-0017] Jendrossek, D. , and Pfeiffer, D. (2014) New insights in the formation of polyhydroxyalkanoate granules (carbonosomes) and novel functions of poly(3‐hydroxybutyrate). Environ Microbiol 16: 2357–2373. 24329995 10.1111/1462-2920.12356

[mbt212820-bib-0018] Keshavarz, T. , and Roy, I. (2010) Polyhydroxyalkanoates: bioplastics with a green agenda. Curr Opin Microbiol 13: 321–326. 20227907 10.1016/j.mib.2010.02.006

[mbt212820-bib-0019] Kuchta, K. , Chi, L. , Fuchs, H. , Pötter, M. , and Steinbüchel, A. (2007) Studies on the Influence of Phasins on Accumulation and Degradation of PHB and Nanostructure of PHB Granules in Ralstonia eutropha H16. Biomacromol 8: 657–662. 10.1021/bm060912e 17291089

[mbt212820-bib-0020] Lan, L. , Zhao, H. , Chen, J. , and Chen, G. (2016) Engineering Halomonas spp. as A Low‐Cost Production Host for Production of Bio‐surfactant Protein PhaP. Biotechnol J 11: 1595–1604. 27687610 10.1002/biot.201600459

[mbt212820-bib-0021] Madison, L.L. , and Huisman, G.W. (1999) Metabolic engineering of poly(3‐hydroxyalkanoates): from DNA to plastic. Microbiol Mol Biol Rev 63: 21–53. 10066830 10.1128/mmbr.63.1.21-53.1999 PMC98956

[mbt212820-bib-0022] Maestro, B. , Galan, B. , Alfonso, C. , Rivas, G. , Prieto, M.A. , and Sanz, J.M. (2013) A new family of intrinsically disordered proteins: structural characterization of the major phasin PhaF from Pseudomonas putida KT2440. PLoS ONE 8: e56904. 23457638 10.1371/journal.pone.0056904 PMC3574117

[mbt212820-bib-0023] Mechin, M.C. , Bertin, Y. , and Girardeau, J.P. (1995) Hydrophobic cluster analysis and secondary structure predictions revealed that major and minor structural subunits of K88‐related adhesins of Escherichia coli share a common overall fold and differ structurally from other fimbrial subunits. FEBS Lett 364: 319–324. 7758589 10.1016/0014-5793(95)00417-8

[mbt212820-bib-0024] Mezzina, M.P. , and Pettinari, M.J. (2016) Phasins, Multifaceted Polyhydroxyalkanoate Granule‐Associated Proteins. Appl Environ Microbiol 82: 5060–5067. 27287326 10.1128/AEM.01161-16 PMC4988190

[mbt212820-bib-0025] Mezzina, M.P. , Wetzler, D.E. , Catone, M.V. , Bucci, H. , Di Paola, M. , and Pettinari, M.J. (2014) A phasin with many faces: structural insights on PhaP from Azotobacter sp. FA8. PLoS ONE 9: e103012. 25077609 10.1371/journal.pone.0103012 PMC4117528

[mbt212820-bib-0026] Mezzina, M.P. , Wetzler, D.E. , de Almeida, A. , Dinjaski, N. , Prieto, M.A. , and Pettinari, M.J. (2015) A phasin with extra talents: a polyhydroxyalkanoate granule‐associated protein has chaperone activity. Environ Microbiol 17: 1765–1776. 25297625 10.1111/1462-2920.12636

[mbt212820-bib-0027] Moldes, C. , Garcia, P. , Garcia, J.L. , and Prieto, M.A. (2004) In vivo immobilization of fusion proteins on bioplastics by the novel tag BioF. Appl Environ Microbiol 70: 3205–3212. 15184113 10.1128/AEM.70.6.3205-3212.2004 PMC427747

[mbt212820-bib-0028] Neumann, L. , Spinozzi, F. , Sinibaldi, R. , Rustichelli, F. , Potter, M. , and Steinbuchel, A. (2008) Binding of the Major Phasin, PhaP1, from Ralstonia eutropha H16 to Poly(3‐Hydroxybutyrate) Granules. J Bacteriol 190: 2911–2919. 18223073 10.1128/JB.01486-07 PMC2293264

[mbt212820-bib-0029] Pfeiffer, D. and Jendrossek, D. (2011) Interaction between poly(3‐hydroxybutyrate) granule‐associated proteins as revealed by two‐hybrid analysis and identification of a new phasin in Ralstonia eutropha H16. Microbiology 157: 2795–2807. 21737497 10.1099/mic.0.051508-0

[mbt212820-bib-0030] Pfeiffer, D. , and Jendrossek, D. (2012) Localization of Poly(3‐Hydroxybutyrate) (PHB) Granule‐Associated Proteins during PHB Granule Formation and Identification of Two New Phasins, PhaP6 and PhaP7, in Ralstonia eutropha H16. J Bacteriol 194: 5909–5921. 22923598 10.1128/JB.00779-12 PMC3486113

[mbt212820-bib-0032] Potter, M. (2004) The complex structure of polyhydroxybutyrate (PHB) granules: four orthologous and paralogous phasins occur in Ralstonia eutropha. Microbiology 150: 2301–2311. 15256572 10.1099/mic.0.26970-0

[mbt212820-bib-0033] Potter, M. , and Steinbuchel, A. (2005) Poly(3‐hydroxybutyrate) granule‐associated proteins: Impacts on poly(3‐hydroxybutyrate) synthesis and degradation. Biomacromol 6: 552–560. 10.1021/bm049401n 15762612

[mbt212820-bib-0034] Potter, M. , Oppermann‐Sanio, F.B. , and Steinbuchel, A. (2001) Cultivation of bacteria producing polyamino acids with liquid manure as carbon and nitrogen source. Appl Environ Microbiol 67: 617–622. 11157224 10.1128/AEM.67.2.617-622.2001 PMC92628

[mbt212820-bib-0035] Prieto, M.A. , Buhler, B. , Jung, K. , Witholt, B. , and Kessler, B. (1999) PhaF, a polyhydroxyalkanoate‐granule‐associated protein of Pseudomonas oleovorans GPo1 involved in the regulatory expression system for pha genes. J Bacteriol 181: 858–868. 9922249 10.1128/jb.181.3.858-868.1999 PMC93452

[mbt212820-bib-0036] Qi, Q. , Steinbuchel, A. , and Rehm, B.H. (2000) In vitro synthesis of poly(3‐hydroxydecanoate): purification and enzymatic characterization of type II polyhydroxyalkanoate synthases PhaC1 and PhaC2 from Pseudomonas aeruginosa. Appl Microbiol Biotechnol 54: 37–43. 10952003 10.1007/s002530000357

[mbt212820-bib-0037] Tian, S. , Lai, W. , Zheng, Z. , Wang, H. , and Chen, G. (2005) Effect of over‐expression of phasin gene from Aeromonas hydrophila on biosynthesis of copolyesters of 3‐hydroxybutyrate and 3‐hydroxyhexanoate. FEMS Microbiol Lett 244: 19–25. 15727816 10.1016/j.femsle.2005.01.020

[mbt212820-bib-0039] Wang, Z. , Wu, H. , Chen, J. , Zhang, J. , Yao, Y. , and Chen, G.Q. (2008) A novel self‐cleaving phasin tag for purification of recombinant proteins based on hydrophobic polyhydroxyalkanoate nanoparticles. Lab Chip 8: 1957–1962. 18941699 10.1039/b807762b

[mbt212820-bib-0040] Wang, Y. , Yin, J. , and Chen, G.Q. (2014) Polyhydroxyalkanoates, challenges and opportunities. Curr Opin Biotechnol 30: 59–65. 24976377 10.1016/j.copbio.2014.06.001

[mbt212820-bib-0041] Wei, D. , Chen, C. , Fang, G. , Li, S. , and Chen, G. (2011) Application of polyhydroxyalkanoate binding protein PhaP as a bio‐surfactant. Appl Microbiol Biotechnol 91: 1037–1047. 21590291 10.1007/s00253-011-3258-7

[mbt212820-bib-0042] Wieczorek, R. , Pries, A. , Steinbuchel, A. , and Mayer, F. (1995) Analysis of a 24‐kilodalton protein associated with the polyhydroxyalkanoic acid granules in Alcaligenes eutrophus. J Bacteriol 177: 2425–2435. 7730274 10.1128/jb.177.9.2425-2435.1995 PMC176901

[mbt212820-bib-0043] Yao, Y. , Zhan, X. , Zhang, J. , Zou, X. , Wang, Z. , Xiong, Y. , *et al* (2008) A specific drug targeting system based on polyhydroxyalkanoate granule binding protein PhaP fused with targeted cell ligands. Biomaterials 29: 4823–4830. 18824258 10.1016/j.biomaterials.2008.09.008

[mbt212820-bib-0044] Zhao, M. , Li, Z. , Zheng, W. , Lou, Z. , and Chen, G. (2006) Crystallization and initial X‐ray analysis of polyhydroxyalkanoate granule‐associated protein from Aeromonas hydrophila. Acta Crystallogr Sect F Struct Biol Cryst Commun 62: 814–819. 10.1107/S1744309106025000 PMC2242923 16880566

[mbt212820-bib-0045] Zhao, H. , Wei, H. , Liu, X. , Yao, Z. , Xu, M. , Wei, D. , *et al* (2016) Structural Insights on PHA Binding Protein PhaP from Aeromonas hydrophila. Sci Rep 6: 39424. 28009010 10.1038/srep39424 PMC5180188

