# Supplementary material for: Modelling of microbial polyhydroxyalkanoate surface binding protein PhaP for rational mutagenesis
Source: Microb Biotechnol. 2017 Aug 25;10(6):1400–11. doi: 10.1111/1751-7915.12820 (PMC5658623; doi:10.1111/1751-7915.12820)
Supplement: Supplementary file 1 — Fig. S1. SDS‐PAGE gels of purified PhaPAz and PhaPTD. Fig. S2. SLS measurements of purified full‐length PhaPTD. Fig. S3. The circular dichroism results of PhaPAz and PhaP TD at 30°C and 100°C. [file MBT2-10-1400-s001.docx]

Supplementary Info

**Modelling of Microbial PHA Surface Binding Protein PhaP for Rational Mutagenesis**

Authors: Hongyu ZHAO^#a^, Zhenyu YAO^#a^, Xiangbin CHEN^a^, Xinquan WANG*^c,d^, Guo-Qiang CHEN*^a,b,e,f^

**Affiliations:**

^a^ Center for Synthetic and Systems Bology, School of Life Sciences, Tsinghua-Peking Center for Life Sciences, Tsinghua University, Beijing 100084, China

^b^ Manchester Institute of Biotechnology, University of Manchester, Oxford Road, Manchester M13 9PT, UK

^c^ MOE Laboratory of Protein Science, Beijing Advanced Innovation Center for Structural Biology, Collaborative Innovation Center for Biotherapy, School of Life Sciences, Tsinghua University, Beijing 100084, China

^d^ Collaborative Innovation Center for Biotherapy, State Key Laboratory of Biotherapy and Cancer Center, West China Hospital, West China Medical School, Sichuan University, Chengdu, China

^e^ Center for Nano and Micro-Mechanics, Tsinghua University, Beijing 100084, China

^f^ MOE Key Lab for Industrial Biocatalysis, Tsinghua University, Beijing 100084, China

^#^Equal contribution

**^*^ Corresponding authors:**

Guo-Qiang CHEN (Chen GQ)

School of Life Sciences, Tsinghua University, Beijing 100084, China

Phone: +86-10-62783844; Fax: +86-10-62794217

E-mail: [chengq@mail.tsinghua.edu.cn](mailto:chengq@mail.tsinghua.edu.cn)

Xinquan WANG (WANG XQ)

School of Life Sciences, Tsinghua University, Beijing 100084, China

E-mail: [xinquanwang@mail.tsinghua.edu.cn](mailto:xinquanwang@mail.tsinghua.edu.cn)


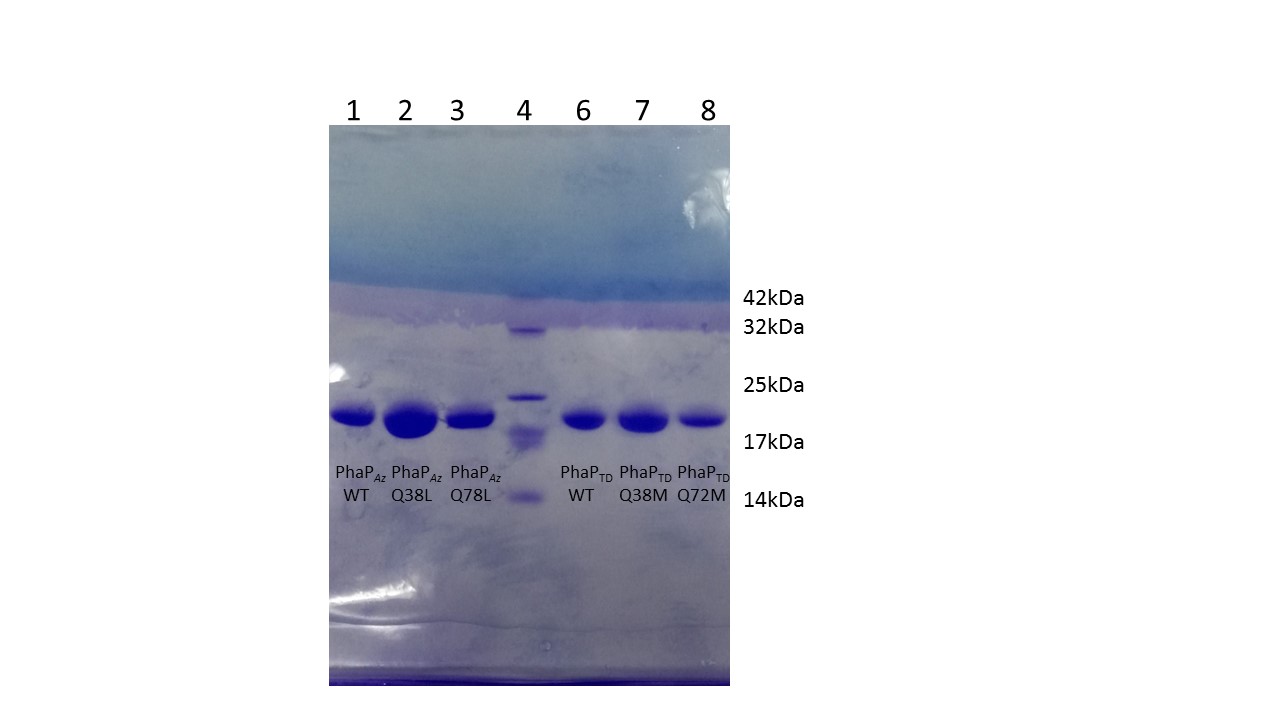


Fig.S1. SDS-PAGE gels of purified PhaP*_Az_* and PhaP*_TD_*

The first lane is the wild type PhaP*_Az,_* the second lane is PhaP*_Az_* Q38L, the third lane is PhaP*_Az_* Q78L, the fourth lane is pageruler prestained protein ladder, the sixth lane is wild type PhaP*_TD_* , the seven lane is PhaP*_TD_* Q38M, the eighth lane is PhaP*_TD_* Q72M.


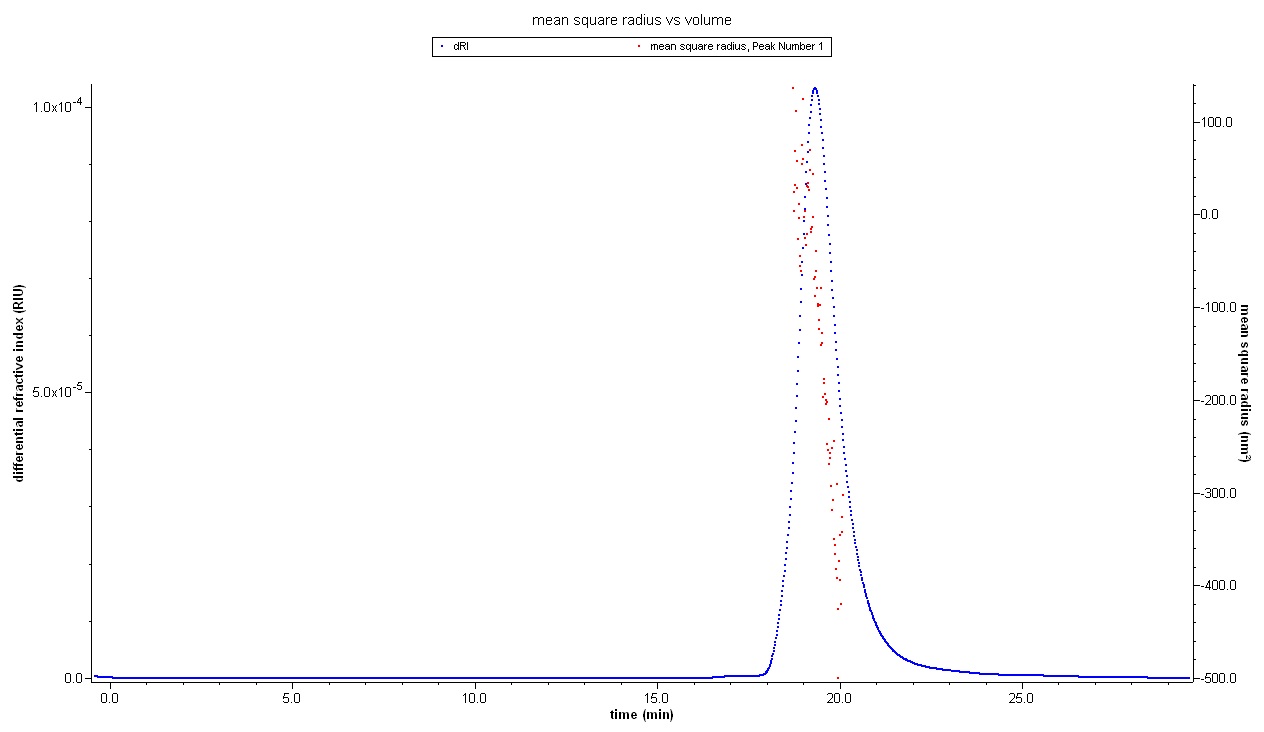


|  | PhaP*_TD_*（full-length） |
| --- | --- |
| Calculated Mass (µg) | 282.57 |
| Mn（Da） | 4.852×10^4^ |
| Mp（Da） | 4.973×10^4^ |
| Mw（Da） | 4.855×10^4^ |

Fig. S2. SLS measurements of purified full-length PhaP*_TD_*

Protein molecular masses were determined via SLS using the DAWN HELEOSTM II eighteen-angle static light-scattering system (Wyatt Technology, USA) connected to a gel-filtration chromatography system equipped with a Superdex 200 high performance column (see above for specifics of gel filtration). The red dot is differential fractive index of PhaP*_TD_* , the blue dot is mean square radius of PhaP*_TD_*. The system was pre-equilibrated with buffer for more than eight hours, and subsequently calibrated with 1 mg/ml BSA. The molecular weight has been shown in the table.


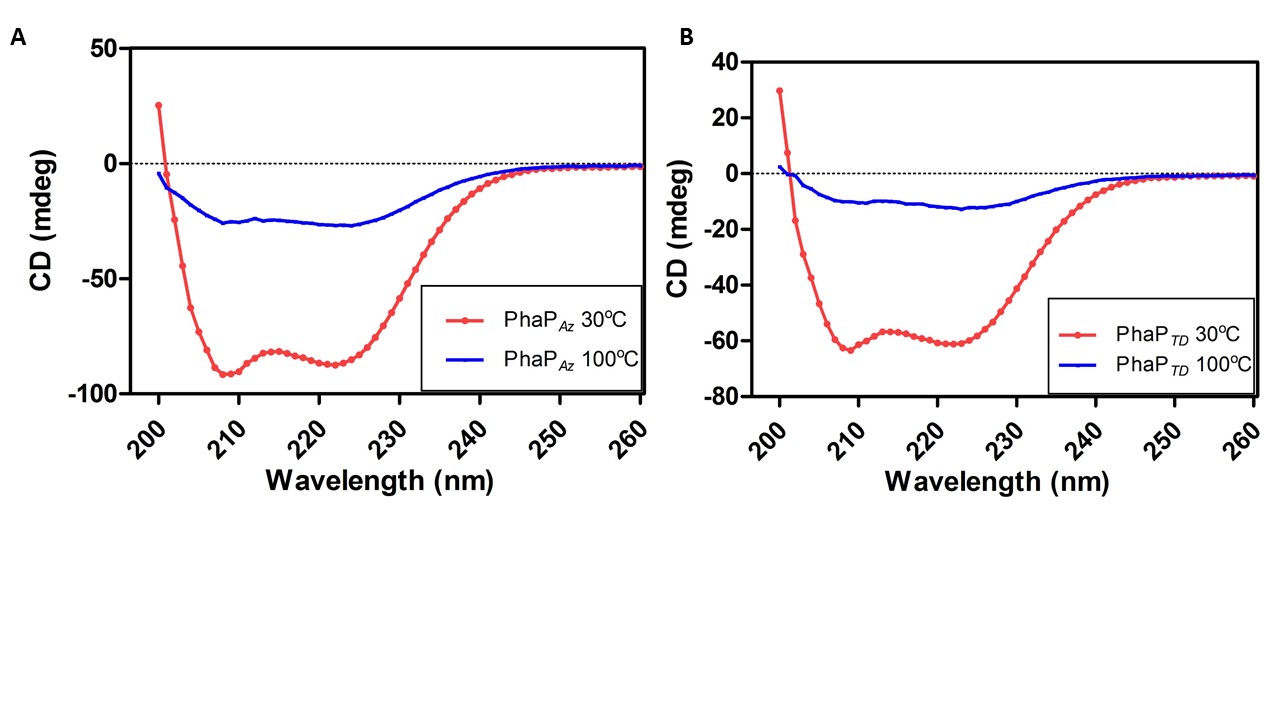


Fig. S3. The circular dichroism results of PhaP*_Az_* and PhaP *_TD_* at 30^o^C and 100^o^C.

These two kinds of proteins were wild type (0.25 mg/ml).
